# Colostrum as a source of ESBL-*Escherichia coli* in feces of newborn calves

**DOI:** 10.1038/s41598-024-60461-4

**Published:** 2024-04-30

**Authors:** Lisa Bachmann, Laura Weber, Wendy Liermann, Harald M. Hammon, Cora Delling, Franziska Dengler, Katharina Schaufler, Michael Schwabe, Elias Eger, Karsten Becker, Anne Schütz, Timo Homeier-Bachmann

**Affiliations:** 1 University of Applied Science Neubrandenburg, Brodaer Str. 2, 17033 Neubrandenburg, Germany; 2 Research Institute of Farm Animal Biology (FBN), Dummerstorf, Germany; 3 Institute for Parasitology, School of Veterinary Medicine, University of Leipzig, Leipzig, Germany; 4 Institute of Physiology, University of Leipzig, Leipzig, Germany; 5 Institute of Physiology, Pathophysiology and Biophysics, University of Veterinary Medicine Vienna, Vienna, Austria; 6 Institute of Pharmacy, University of Greifswald, Greifswald, Germany; 7 Department Epidemiology and Ecology of Antimicrobial Resistance, Helmholtz Centre for Infection Research, Helmholtz Institute for One Health, Greifswald, Germany; 8 Institute of Infection Medicine, Christian-Albrecht University Kiel and University Medical Center Schleswig-Holstein, Kiel, Germany; 9 Friedrich Loeffler-Institute of Medical Microbiology, University Medicine Greifswald, Greifswald, Germany; 10 Institute of Epidemiology, Friedrich-Loeffler-Institute, Greifswald, Insel Riems Germany

**Keywords:** Antimicrobial resistance, Phylogeny, DNA sequencing

## Abstract

The aim of the present study was to determine if colostrum and the equipment for harvesting and feeding colostrum are sources of fecal ESBL/AmpC-producing *Escherichia coli* (ESBL/AmpC-*E. coli*) in calves. Therefore, 15 male calves fed with pooled colostrum on a dairy farm and held individually in an experimental barn, the colostrum pool and the equipment for harvesting and feeding colostrum were sampled and analyzed for the occurrence of ESBL/AmpC-*E. coli.* The ESBL-AmpC-*E. coli* suspicious isolates were subjected to whole-genome sequence analysis*.* Forty-three of 45 fecal samples were tested positive for ESBL/AmpC-*E. coli*. In the colostrum sample and in the milking pot, we also found ESBL/AmpC-*E. coli.* All 45 *E. coli* isolates were ESBL-producers, mainly commensal sequence type (ST) 10, but also human-extraintestinal pathogenic *E. coli* ST131 and ST117 were found. The clonal identity of six fecal isolates with the ESBL-*E. coli* isolate from the colostrum and of five fecal isolates with the strain from the milking pot demonstrates that the hygiene of colostrum or the colostrum equipment can play a significant role in the spread of ESBL-*E. coli*. Effective sanitation procedures for colostrum harvesting and feeding equipment are crucial to reduce the ESBL-*E. coli* shedding of neonatal dairy calves.

## Introduction

In 2015, the World Health Organization (WHO) designated antimicrobial resistance (AMR) as a major threat to global health, food security, and development. Extended-spectrum β-lactamase (ESBL)-producing bacteria are resistant to broad-spectrum β-lactams, such as 3rd generation cephalosporins. Recognizing the severity of this resistant phenotype in terms of increasing mortality rates in humans and animals, ESBL-producing *Enterobacterales* have been included in the WHO global priority list of antibiotic-resistant bacteria, which guides research for the discovery and development of new antibiotics^1,2^. Apart from ESBL, resistance to 3rd generation-cephalosporins can also be mediated through AmpC β-lactamases (AmpC)^3^.

ESBL/AmpC-producing *Enterobacterales*, especially *Escherichia coli* (*E. coli*), are frequently found in livestock (e.g., dairy cattle), as well as in food products^4–6^. Although commensal *E. coli* strains rarely cause infections, they can, however, transfer resistance genes horizontally to pathogenic *E. coli* strains or other *Enterobacterales* that can be transmitted to humans via the food chain or environmental effluents^7–9^.

Usually, the prevalence of ESBL/AmpC-producing *E. coli* (ESBL/AmpC-*E. coli*) in dairy cattle is typically age-dependent with a higher prevalence and abundance in pre-weaning calves^10^. A recent study in Germany showed that 63.5% of the young calves in large dairies shed ESBL/AmpC-*E. coli* although most of them had never been treated with antibiotics^11^. With increasing age and dietary transformation from a monogastric animal to a ruminant, ESBL/AmpC-*E. coli* excretion decreases^12^. This indicates that diet is a strong contributing factor of the fecal release of antibiotic-resistant bacteria^13^.

There are several studies investigating risk factors for the occurrence of ESBL/AmpC-*E. coli* in calves^11,14–17^. Concerning diet, some of them could reveal that feeding of waste milk containing antibiotic residues increases ESBL/AmpC-*E. coli* prevalence in calves^11,14,18^. Another study associated the antibiotic dry-off therapy of the cows with higher fecal ESBL/AmpC-*E. coli* shedding in calves, suggesting an influence of antibiotic residues in colostrum on ESBL/AmpC-*E. coli* occurrence^19^. However, in addition to antibiotic contamination of colostrum or milk rations, vertical transmission of ESBL/AmpC-*E. coli* from the dam, horizontal transmission from other animals in the herd, or transmission from the housing environment, colostrum/feed, or feeding equipment may also play an important role^11,13,16,20^.

As calves are the main contributors of ESBL/AmpC-*E. coli* release in dairy production, understanding the early colonization of ESBL/AmpC-*E. coli* in calves during the milk-feeding period is of great importance. As part of a concurrent study, in which calves were fed with pooled colostrum, we could sample feces, colostrum and the equipment for harvesting and feeding colostrum to get a deeper insight in the sources for the early acquisition of ESBL/AmpC-*E. coli*. According to the research of Liu et al.^13^ and of He et al.^21^ detecting the same genes for AMR in colostrum and calves’ feces, we hypothesized that colostrum or the equipment may serve as the first vectors for ESBL/AmpC-*E. coli* in young calves.

## Results

### Bacteriological examination

On the first and fifth day of life, 14 out of 15 fecal samples of the calves were tested positive for ESBL/AmpC-*E. coli*. On day 8 all calves showed ESBL/AmpC-*E. coli* excretion. Furthermore, we detected ESBL/AmpC-*E. coli* in the colostrum sample and in the swab of the milking pot. In total, we obtained 45 ESBL-*E. coli* isolates (43 from feces and one from colostrum and one from equipment for harvesting colostrum).

### Antimicrobial susceptibility test (AST)

The results of the AST are displayed in Table 1. All 45 ESBL/AmpC-suspicious *E. coli* isolates showed ESBL- and not AmpC-phenotype, i.e., were resistant against ampicillin, piperacillin, cefuroxime, cefotaxime and ceftazidime, but were less resistant against combinations of β-lactam antibiotics with inhibitors of β-lactamases (tazobactam, avibactam). Twenty-four (= 53.3%) of the isolates fulfilled the definition of multidrug-resistant (MDR, resistant to at least three antibiotic classes) organisms. Of the MDR isolates, 14 (= 31% of all isolates) showed phenotypic resistance against ciprofloxacin. All isolates were sensitive to ertapenem, imipenem, meropenem, gentamicin, eravacycline, tigecycline, and colistin.

**Table 1:** Phenotypic resistance profiles of ESBL-*E. coli* isolates. *R* resistant, *S* sensitive, *I* intermediate.

Designation	Calf No.	Day of sampling	ESBL	MDR	Ampicillin	Ampicillin/sulbactam	Piperacillin	Piperacillin/tazobactam	Cefuroxime	Cefuroxime axetil	Cefotaxime	Ceftazidime	Ceftazidime/avibactam
442	1	1	+	−	R	R	R	I	R	R	R	R	S
443	1	5	+	+	R	R	R	I	R	R	R	R	S
444	1	8	+	+	R	R	R	I	R	R	R	R	S
445	2	1	+	−	R	R	R	I	R	R	R	R	S
446	2	5	+	−	R	R	R	I	R	R	R	R	S
447	2	8	+	−	R	R	R	I	R	R	R	R	S
448	3	1	+	+	R	R	R	I	R	R	R	R	S
449	3	5	+	+	R	R	R	I	R	R	R	R	S
450	3	8	+	−	R	R	R	I	R	R	R	R	S
451	4	1	+	−	R	R	R	I	R	R	R	R	S
452	4	5	+	+	R	R	R	I	R	R	R	R	S
453	4	8	+	−	R	R	R	I	R	R	R	R	S
460	5	1	+	−	R	R	R	I	R	R	R	R	S
461	5	5	+	+	R	R	R	I	R	R	R	R	S
462	5	8	+	−	R	R	R	I	R	R	R	R	S
463	6	5	+	+	R	R	R	I	R	R	R	R	S
464	6	8	+	+	R	R	R	I	R	R	R	R	S
465	7	1	+	+	R	R	R	I	R	R	R	R	S
454	7	5	+	+	R	R	R	I	R	R	R	R	S
455	7	8	+	−	R	R	R	I	R	R	R	R	S
466	8	1	+	−	R	R	R	I	R	R	R	R	S
456	8	5	+	−	R	R	R	I	R	R	R	R	S
457	8	8	+	−	R	R	R	I	R	R	R	R	S
912	9	1	+	−	R	R	R	I	R	R	R	R	S
913	9	5	+	+	R	R	R	I	R	R	R	R	S
914	9	8	+	+	R	R	R	I	R	R	R	R	S
915	10	1	+	−	R	R	R	I	R	R	R	R	S
916	10	5	+	+	R	R	R	R	R	R	R	R	R
917	10	8	+	+	R	R	R	I	R	R	R	R	S
918	11	1	+	−	R	R	R	I	R	R	R	R	S
919	11	5	+	+	R	R	R	I	R	R	R	R	S
920	11	8	+	+	R	R	R	I	R	R	R	R	S
921	12	1	+	+	R	R	R	I	R	R	R	R	S
922	12	8	+	+	R	R	R	I	R	R	R	R	S
923	13	1	+	+	R	R	R	R	R	R	R	R	R
924	13	5	+	−	R	R	R	I	R	R	R	R	S
925	13	8	+	−	R	R	R	I	R	R	R	R	S
2009	14	1	+	+	R	R	R	I	R	R	R	R	S
2010	14	5	+	−	R	R	R	I	R	R	R	R	S
2014	14	8	+	−	R	R	R	I	R	R	R	R	S
2018	15	1	+	+	R	R	R	I	R	R	R	R	S
2017	15	5	+	+	R	R	R	I	R	R	R	R	S
2005	15	8	+	+	R	R	R	I	R	R	R	R	S
996	Colostrum		+	−	R	R	R	I	R	R	R	R	S
2012	Milk. pot		+	+	R	R	R	I	R	R	R	R	S

Designation	Ceftolozane/tazobactam	Ertapenem	Imipenem	Meropenem	Imipenem/cilastatin/relebactam	Meropenem/vaborbactam	Gentamicin	Ciprofloxacin	Eravacycline	Tigecycline	Colistin	Trimethoprim-sulfamethoxazole	
442	S	S	S	S	S	S	S	S	S	S	S	S	
443	S	S	S	S	S	S	S	R	S	S	S	R	
444	S	S	S	S	S	S	S	I	S	S	S	R	
445	S	S	S	S	S	S	S	I	S	S	S	S	
446	S	S	S	S	S	S	S	I	S	S	S	S	
447	S	S	S	S	S	S	S	I	S	S	S	S	
448	S	S	S	S	S	S	S	I	S	S	S	R	
449	S	S	S	S	S	S	S	R	S	S	S	S	
450	S	S	S	S	S	S	S	I	S	S	S	S	
451	S	S	S	S	S	S	S	I	S	S	S	S	
452	S	S	S	S	S	S	S	I	S	S	S	R	
453	S	S	S	S	S	S	S	I	S	S	S	S	
460	S	S	S	S	S	S	S	S	S	S	S	S	
461	S	S	S	S	S	S	S	R	S	S	S	R	
462	S	S	S	S	S	S	S	I	S	S	S	S	
463	S	S	S	S	S	S	S	R	S	S	S	S	
464	S	S	S	S	S	S	S	R	S	S	S	R	
465	R	S	S	S	S	S	S	R	S	S	S	R	
454	S	S	S	S	S	S	S	R	S	S	S	S	
455	S	S	S	S	S	S	S	I	S	S	S	S	
466	R	S	S	S	S	S	S	I	S	S	S	S	
456	S	S	S	S	S	S	S	S	S	S	S	S	
457	S	S	S	S	S	S	S	I	S	S	S	S	
912	S	S	S	S	S	S	S	S	S	S	S	S	
913	R	S	S	S	S	S	S	I	S	S	S	R	
914	R	S	S	S	S	S	S	R	S	S	S	R	
915	S	S	S	S	S	S	S	S	S	S	S	S	
916	R	S	S	S	S	S	S	R	S	S	S	R	
917	R	S	S	S	S	S	S	R	S	S	S	R	
918	S	S	S	S	S	S	S	I	S	S	S	S	
919	S	S	S	S	S	S	S	R	S	S	S	R	
920	S	S	S	S	S	S	S	R	S	S	S	R	
921	S	S	S	S	S	S	S	I	S	S	S	R	
922	S	S	S	S	S	S	S	R	S	S	S	S	
923	R	S	S	S	S	S	S	R	S	S	S	R	
924	S	S	S	S	S	S	S	S	S	S	S	S	
925	S	S	S	S	S	S	S	S	S	S	S	S	
2009	S	S	S	S	S	S	S	I	S	S	S	R	
2010	S	S	S	S	S	S	S	I	S	S	S	S	
2014	S	S	S	S	S	S	S	S	S	S	S	S	
2018	S	S	S	S	S	S	S	I	S	S	S	R	
2017	S	S	S	S	S	S	S	I	S	S	S	R	
2005	S	S	S	S	S	S	S	I	S	S	S	R	
996	S	S	S	S	S	S	S	I	S	S	S	S	
2012	S	S	S	S	S	S	S	I	S	S	S	R	

### Whole genome sequencing (WGS)

Details of the WGS are shown in Table 2. MLS typing revealed 13 different sequence types (STs), belonging to ST10, ST88, ST117, ST131, ST354, ST362, ST540, ST744, ST761, ST1122, ST1429, ST1431, and ST2325, with ST10 being dominant in the fecal swabs (21/43 = 49%). Moreover, the isolates of colostrum and milking pot belonged to ST10. The most frequent STs besides ST10 were ST88, ST117 and ST362, each occurring three times. Based on sequencing, ESBL genes (CTX-M type) were detected in all 45 isolates. Most isolates harbored *bla*_*CTX-M-1*_ (35/45 = 78%), whereas we could also detect *bla*_*CTX-M-15*_ (8/45) and *bla*_*CTX-M-27*_ (5/45)*,* thus, three isolates were positive for both*, bla*_*CTX-M1*_ and *bla*_*CTX-M27*_*.* Other β-lactamase genes present in the samples were: *bla*_*oxa-1*_ (8/45), *bla*_*lap-2*_ (9/45) and *bla*_*TEM-1*_ (25/45)*. qnrS1* gene, which is associated with quinolone resistance, was determined in 19 isolates including the ESBL-*E. coli* isolated from colostrum and milking pot. The most common resistance gene was *mdf* (41/45), which belongs to a transport protein conveying resistance to a broad spectrum of toxic substances and antibiotics. Some of the isolates additionally harbored phenicol resistance genes (*floR*, *catA* and *cmlA*). In addition, several aminoglycoside, tetracycline, sulfonamide and trimethoprim, and macrolide resistance genes were also found in the WGS analysis.

**Table 2:**
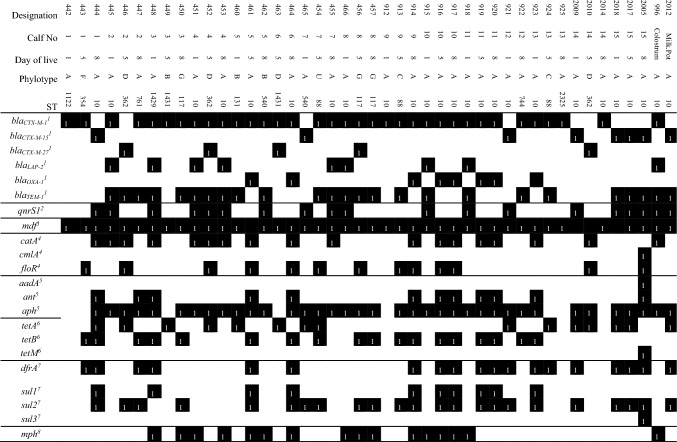
Genotypic characterization of sequenced ESBL-*E. coli* isolates; presence of a certain factor is based on the results from ABRicate [ABRicate v. 1.0.0 (https://github.com/tseemann/abricate), databases used: VFDB, ResFinder, PlasmidFinder, BacMet, ARG-ANNOT, and Ecoli_VF] using de novo-assembled sequences and is depicted in black. Detected genes are assigned to the following categories: ^1^β-lactam antibiotics, ^2^quinolone, ^3^multidrug resistance translocators, ^4^phenicol antibiotics, ^5^aminoglycosides, ^6^tetracycline antibiotics, ^7^sulfonamides and trimethoprim, and ^8^macrolide, lincosamide, and streptogramin B.

### Clonal expansion

**Figure 1:**
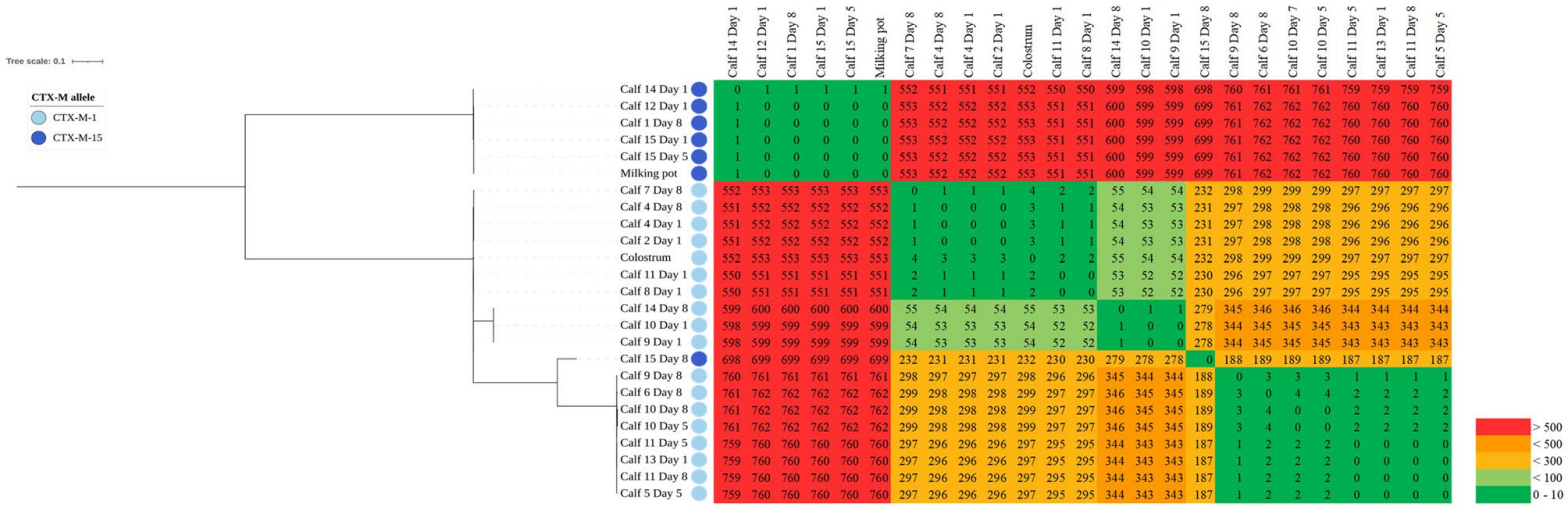
Core SNP phylogeny of ESBL-producing ST10 isolates originating from calf feces, pooled colostrum, and milking pot. Core genome SNPs were called using snippy v. 4.4.1. SNP distances were calculated and depicted in a heat diagram (SNP distance ranged from 0 to 760 sites). The phylogenetic tree is based on a core SNP alignment and visualized in iTOL. Additionally, presence of CTX-M-1 and CTX-M-15 were depicted in the phylogenetic tree.

Phylogeny analysis of ST10 isolates based on WGS data revealed three phylogenetic clusters. The size of the core genome of the ST 10 isolates studied was 5,140,627 bp and within each cluster core genomes of the isolates differed only in 0–4 SNPs or in 0–55 SNPs, respectively (see Fig. 1). The isolate No. 2012 (swab of milking pot) belonged to one cluster, in which only the fecal isolate No. 2009 differed in a single SNP from the other five fecal isolates. All isolates of that milking pot-cluster harbored the CTX-M-15 gene. To the second cluster the isolate No. 996 (colostrum sample) was allocated differing only in 2–4 SNPs to six fecal isolates. Three additional ESBL-*E. coli* isolates of that cluster exhibited more varying SNPs (52–55) compared to the other seven isolates. CTX-M-1 was detected in all isolates of the colostrum-cluster. The third cluster only contained fecal isolates which differed in 0–4 SNPs from each other. In the feces-cluster CTX-M-1 gene was present. The fecal isolate No. 2005 could not be allocated to one of the clusters. The difference to all other isolates of ST10 was 187–699 SNPs. In contrast to the isolates nearby in the phylogenetic tree, this isolate was CTX-M-15 positive.

### Biofilm formation

**Figure 2:**
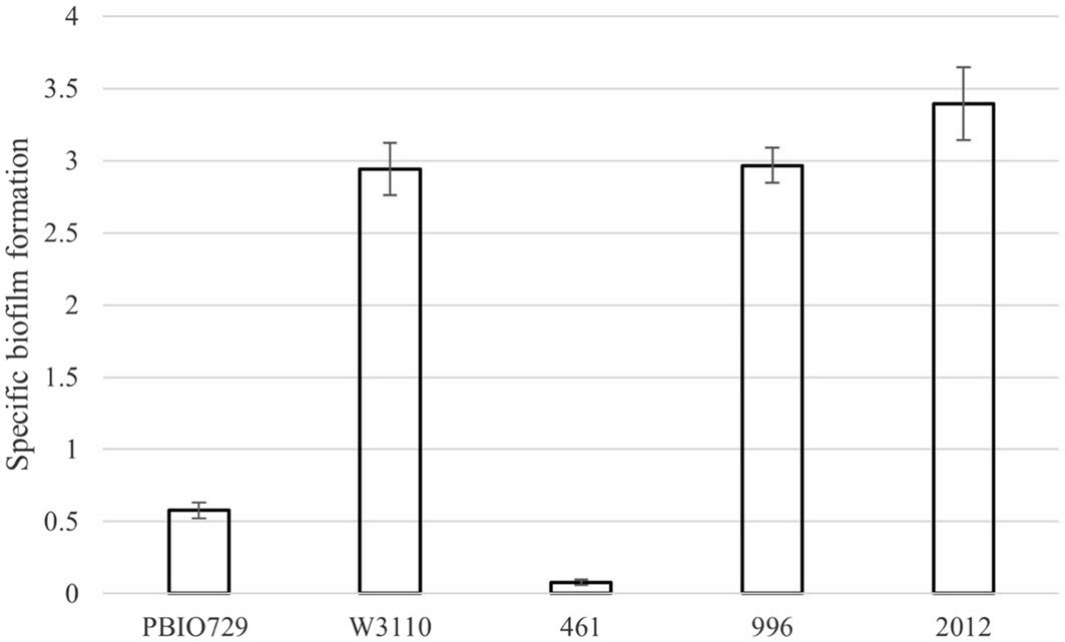
Biofilm formation (averaged mean ± standard deviation) of isolate No. 461 (feces), 996 (colostrum) and 2012 (milking pot) in comparison to PBIO729 (weak biofilm former) and W3110 (strong biofilm former).

To test the ability to form biofilms, one representative was selected from each of the three different clusters we identified in the clonal expansion analysis: No. 2012 (milking pot isolate), No. 996 (colostrum isolate), and No. 461 (fecal isolate). The test showed that the ability to form specific biofilms is high in the isolates from milking pot and colostrum and low in the fecal isolate from the feces-cluster (see Fig. 2).

## Discussion

Today, livestock production is known to be a significant source of AMR. In the past decades, the occurrence of ESBL-carrying pathogens increased worldwide^22^. ESBL/AmpC-*E. coli* are less prevalent in dairy cattle than other livestock species, with the exception of suckling calves, which have a high ESBL/AmpC-*E. coli* prevalence, e.g., above 50% in Germany^11,17^.

Due to their immature immune system, newborn calves depend on passive immunization with maternal antibodies from the colostrum^23^. As it is common practice in conventional dairy farms to separate calf and dam right after birth, the colostrum is usually administered to the calf via bottle, bucket, or drench. A major advantage of this method compared to natural intake is the controllability of the amount and timing of colostrum intake.

Already on the first day of life, 14 out of 15 calves in our study were tested positive for ESBL-*E. coli*, although they had neither spatial nor temporal contact with each other and did not get any antibiotics. Before the next calf could enter the experimental barn, it was cleaned, disinfected and unoccupied for at least 1 week to prevent nosocomial transmission. Therefore, horizontal transmission between the calves and from the equipment of the experimental barn seemed unlikely. Moreover, the transport vehicle has also been cleaned and disinfected, so that transmission of ESBL-*E. coli* during transportation is also unlikely. Moreover, according to previous research, there is little chance that transmission from the dams to the calves is the main reason for ESBL-*E. coli* occurrence in the calves as the prevalence of the corresponding dams is quite low^11^ and the ESBL-*E. coli* strains of calves differ from that of older animals^10^. Therefore, a sample of the colostrum pool and swabs of the equipment for harvesting and administering colostrum were analyzed for the occurrence of ESBL/AmpC-*E. coli*. In colostrum and in the milking pot for harvesting colostrum, we could detect ESBL-*E. coli* belonging to commensal ST10.

WGS revealed that six of 43 fecal isolates were nearly identical to the colostrum strain. Since the number of core genome SNPs was below 17, these isolates fulfil the clone definition of Ludden et al.^24^ who developed this SNP cut-off to demonstrate transmission between patients. An even higher degree of core genomic identity was also demonstrated between five fecal isolates and the milking pot strain, demonstrating direct transmission of ESBL *E. coli* via colostrum or equipment in a total of 11 of the 43 ESBL *E. coli* isolates (25.6%). Our findings are underlined by two recent studies, which assumed that the feeding of colostrum contaminated with ESBL-*E. coli* can be responsible for intestinal colonization of calves with ESBL-*E. coli*, as the same bacterial resistome (i.e., all AMR genes and their precursors) was found in colostrum and in feces of neonatal calves^13,21^. In addition to the suggestion that antibiotic residues in colostrum may lead to increased occurrence of ESBL-*E. coli* in suckling calves^19^, our results prove that colostrum serves as an early vector for resistant bacteria in calves.

It remains unknown how exactly the ESBL-*E. coli* contamination of the colostrum occurred. However, based on the detection of an ESBL-*E. coli* in the milking pot, we assume that the presence of ESBL *E. coli* in the colostrum is due to poor hygiene of the milking equipment used to collect colostrum, particularly since both strains found in colostrum and milking pot have a high ability to form specific biofilms. The ability of biofilm formation allows ESBL *E. coli* to withstand standard cleaning procedures^8^ and to persist in the milking equipment. In this way, washing out bacteria results in contamination of colostrum and colonization of calves. According to two German studies, hygiene management of the calf’s feeding equipment is associated with the occurrence of ESBL-*E. coli* in dairy farms. Heinemann and colleagues found ESBL-*E. coli* in the inner surface of nipples of feeding buckets and concluded that sanitation measures in dairy farms are inadequate, maybe leading to ESBL-*E. coli* infection of the calves^20^. In addition, the cleaning procedure of feeding buckets was associated with the ESBL-E. *coli* prevalence of dairy calves^11^.

Acidification or pasteurization of colostrum, which are common to optimize hygiene in milk rations for calves, are possible methods to sanitize colostrum^25,26^. However, if the equipment for colostrum administration, particularly the nipple, is contaminated with biofilm forming ESBL-*E. coli*, transmission of resistant bacteria is still possible. According to the data of Heinemann et al. ^20^ nipples for calves are regularly contaminated with ESBL-*E. coli* and other bacteria.

In addition to ST10, we found other STs of ESBL-producing *E. coli*, including ST131, a globally distributed clonal lineage known to cause severe extraintestinal infections in humans and animals^22,27^. This single lineage is mainly responsible for the increase in urinary tract and bloodstream infections with ESBL-*E. coli* worldwide. Strains of ST131 usually carry *bla*_CTX-M-15_ on a plasmid^26,28^. Deviating from that, our isolate of ST131 is *bla*_CTX-M-1_-positive. ESBL-*E. coli* carrying *bla*_CTX-M1_ are primarily isolated from livestock in Europe^29,30^. CTX-M15 is predominant in ST131^29,31^, therefore, horizontal gene transfer of a *bla*_*CTX-M-1*_ encoding plasmid seems certainly probable for our isolate. In studies with companion animals, ESBL-*E. coli* producing CTX-M-15 and other variants (e.g., CTX-M-14, CTX-M-55), which were frequently found in humans, could be detected, indicating a direct transfer of viable bacteria between companion animals and humans^30,32^. Transmission of ESBL-*E. coli* from animals to humans is often postulated. Regarding livestock production, contaminated slurry and waste water from abattoirs and animal rendering plants can harbor a significant risk for human health^8,33,34^.

Among our study, we also detected ST88, ST117 and ST362, which seem to be associated with calves as they were also found in a farm in Mecklenburg Western Pomerania^10^. ST362 is also known to be an efficient biofilm former^33,35^, therefore, is able to survive at surfaces of milking or feeding equipment or at calf barns/igloos^10,20^. Just like ST131, ST117 is also related to extraintestinal infections in humans and results of five studies indicate that poultry may be the reservoir, but it could also be found in calves^34,36^. With the present study being at least the third detecting ST117 in calves, it cannot be denied that calves may also play a role as a vector for human or avian ST117 colonization.

To our concern, > 50% of all isolates fulfilled the definition of being MDR and 31% of the ESBL-*E. coli* showed phenotypic quinolone resistance. Fortunately, none of the strains associated with human infections harbored multiple AMR.

In the present study, we were able to detect ESBL *E. coli* in a large number of different STs, which underlines the fundamental need for improved hygiene in calf husbandry. The transmission modes of the individual STs must be the subject of further investigations. With regard to colostrum as a possible source of ESBL *E. coli*, we were able to demonstrate the direct transmission of viable ESBL-*E. coli* via contaminated colostrum. Besides lowering the use of antibiotic agents in dairy herds, excellent sanitation procedures of the equipment for harvesting and feeding colostrum is crucial to reduce the prevalence of ESBL-*E. coli* in neonatal dairy calves, consequently minimizing the spread of AMR. Therefore, more attention should be paid on the improvement of farm hygiene, as it remains to be a simple and efficient method to prevent the environmental contamination with ESBL-*E. coli.*

## Material and methods

### Animals

Fecal, colostrum and equipment samples were collected as part of another study^37^ in which calves were initially fed with pooled colostrum at birth and thereafter moved to an experimental barn where they were individually housed and fed with milk replacer within the first eight days of life. Briefly, 15 male Holstein–Friesian calves from a German dairy farm were fed 3 L of colostrum right after birth at the farm. The colostrum was pooled prior to the beginning of the experiments and stored frozen in portions so that all calves received the same colostrum. The calves were then transported to the University of Leipzig within their first 24 h of life. There, they were stalled in one of two separate barns (control and infected with *Cryptosporidium parvum*, see below) with only one calf at a time. The calves were fed 3 × 2 L of milk replacer (Union A50 S, Arla Foods, Viby J, Denmark) daily and had free access to water in a bucket. Additionally, from day 4 onward they received 2 L of electrolytes (Ursolyt G oral, Serumwerk Bernburg AG, Bernburg, Germany) daily.

Upon arrival at the University, the animals were examined clinically, and blood and fecal samples were collected. Afterwards, one group of calves was infected by oral application of 2 × 10^7^ *Cryptosporidium parvum* oocysts (n = 5) whereas the control group received only water. In the following days, the calves underwent several blood and fecal samplings for study purposes according to the aims of the concurrent study^37^.

The experiments, including all animal sampling, were conducted in accordance with the German legislation on the protection of animals and were approved by the Landesdirektion Leipzig as TVV 19/20. All authors comply with the ARRIVE guidelines.

### Sampling, bacteriological examination

Fecal swabs (Sigma Transwab, MWE, United Kingdom) for the determination of ESBL/AmpC-*E. coli* carriage of the calves were taken at the 1st day (before infection with *C. parvum*), day 5 and day 8 of life, preserved in Amies medium and stored at 5 °C. A sample of the stored colostrum pool was additionally analyzed, and subsequently the dairy farm was visited and the equipment for harvesting and administering colostrum including liner, tube and milking pot and the nipple of the colostrum bottle was sampled using swabs.

Fecal, colostrum and equipment samples were cultured on CHROM ID agar plates (Mast Group, Reinfeld, Germany) supplemented with 2 µg/mL cefotaxime (Alfa Aesar by Thermo Fisher Scientific, Kandel, Germany) and incubated at 37 °C overnight. Moreover, the pooled colostrum was enriched in LB-broth supplemented with 2 µg/mL cefotaxime before cultivation. According to the manufacturer’s protocol, pink-violet colored, shiny colonies represent presumptive ESBL/AmpC-*E. coli*-positive results. Positive colonies were picked and sub-cultivated until a pure culture was achieved. All isolates were stored at − 80 °C until further use.

### Antimicrobial susceptibility testing

AST was carried out using VITEK2 (bioMérieux, Nürtingen, Germany). Testing was performed using software version 9.02 and AST-N428 and AST-XN24 card, according to the manufacturer’s instructions. The AST card used for the VITEK2 included an ESBL confirmation test. Second and 3rd generation cephalosporins (ceftazidime, cefotaxime and cefuroxime) were used alone or in combination with tazobactam/avibactam. A reduction of growth in the presence of inhibitor of β-lactamases was considered indicative of ESBL production.

Minimal inhibitory concentration (MIC) breakpoints were set according to the European Committee on Antimicrobial Susceptibility Testing (EUCAST) breakpoint tables for interpretation of MICs and zone diameters (Version 13.1, 2023. http://www.eucast.org).

### Whole genome sequencing and analysis

Whole genome sequencing of all isolates was applied (n = 45). DNA extraction was performed using the MasterPure™ DNA Purification Kit for Blood, Version II (Lucigen, Middleton, USA) and subsequently quantified using a Qubit 4 fluorometer (Thermofisher Scientific, Waltham, USA). DNA samples were then shipped to the Microbial Genome Sequencing Center (MiGS, Pittsburgh, PA, USA). Sample libraries were prepared using the Illumina DNA Prep kit and IDT 10 bp UDI indices, and sequenced on an Illumina NextSeq 2000, producing 2 × 151 bp reads. Demultiplexing, quality control and adapter trimming was performed with bcl-convert (v3.9.3) (Illumina, Inc.; https://support-docs.illumina.com/SW/BCL_Convert/Content/SW/FrontPages/BCL_Convert.htm).

The sequence analysis is described elsewhere^38,39^. In brief: We used BBDuk from BBTools v. 38.89 (http://sourceforge.net/projects/bbmap/) for (i) adapter-trimming, (ii) filtering for contaminants, and (iii) quality-trimming. For de novo genome assembly we used the shovill v. 1.1.0 assembly pipeline (https://github.com/tseemann/shovill) in combination with SPAdes v. 3.15.0^40^. Thereafter, assemblies were analyzed for multilocus sequence type (MLST) determination and antibiotic resistance/virulence gene detection using the tools mlst v. 2.19.0 (https://github.com/tseemann/mlst) and ABRicate v. 1.0.0 (https://github.com/tseemann/abricate), respectively. Third-party databases (e.g., PubMLST^41^, VFDB^42^, ResFinder^43^, PlasmidFinder^44^, BacMet^45^, ARG-ANNOT^46^, and Ecoli_VF (https://github.com/phac-nml/ecoli_vf)) were used for the analyses of both tools.

### Clonal expansion

To create the core SNP phylogeny of the 25 ESBL-producing ST10 isolates, the reads were mapped against 919 as reference genome using Snippy v. 4.6.0 (https://github.com/tseemann/snippy, accessed on 12.08.2022) to generate an alignment of these sequences. In the next step, the alignment was processed using Gubbins v.2.4.1^47^, snp-sites v. 2.5.1^48^ and FastTree v. 2.1.11 (http://www.microbesonline.org/fasttree/) (in detail described elsewhere^5^). The final alignment was midpoint-rooted in iTOL v. 6.7.2^49^ and vizualized with the CTX-M types.

Snp-dists v. 0.8.2. (https://github.com/tseemann/snp-dists, accessed on 05.06.2023) was used to convert the FASTA alignment to a SNP distance matrix. (The distance matrix was depicted as a heat map diagram beside the core SNP phylogeny.)

### Biofilm formation

Biofilm formation on polystyrene surfaces was assessed using crystal violet (CV) staining, as previously described^5^. The strength of biofilm formation was determined as specific biofilm formation (SBF), which was calculated using the formula: SBF = (B − NC)/G, where B is the OD570 of the stained bacteria, NC is the OD570 of the stained control wells to account for CV adhering to the polystyrene surface due to non-biological factors, and G is the OD600 representing the cell density in the culture medium. To evaluate biofilm formation, the test was also performed with two strains which are weak (PBIO729^50^) or strong (W3110) biofilm formers, respectively.

## Data Availability

The datasets generated during and/or analysed during the current study are available from the corresponding author on reasonable request.
